# Quantitation of circulating wild-type alpha-1-antitrypsin in heterozygous carriers of the S and Z deficiency alleles

**DOI:** 10.1186/s12931-015-0256-9

**Published:** 2015-08-05

**Authors:** L. J. Donato, R. M. Karras, J. A. Katzmann, D. L. Murray, M. R. Snyder

**Affiliations:** Department of Laboratory Medicine & Pathology Mayo Clinic, Mayo Clinic, 200 First St. SW, 55905 Rochester, MN USA; Present address: University of Minnesota, Minneapolis, MN USA

## Abstract

**Background:**

Alpha-1-antitrypsin (A1AT) deficiency disease results from mutations in the A1AT gene. Controversy exists in regards to treatment of heterozygous carriers of the S and Z deficiency alleles. Quantitation of allelic expression has not been possible with standard laboratory methods. Here we show that the recently described method for liquid chromatography tandem mass spectrometry (LC-MS/MS) analysis of A1AT tryptic peptides can differentiate between mutated (S and Z) and wild-type (non-S and non-Z) proteins allowing for quantitation of circulating allelic expression in heterozygous patients.

**Methods:**

Serum (244 M/M, 61 M/Z, and 63 M/S) was combined with isotopically labeled peptide standards, digested with trypsin, and quantitated by LC-MS/MS. Total and allele-specific A1AT quantitation was performed by comparison of peptide peak height ratios to a standard curve for each peptide. Linear regression was used to compare results and central 95^th^ percentile intervals were calculated using parametric analysis.

**Results:**

Quantitation of circulating wild-type A1AT based on the proteotypic and allelic (non-S and non-Z) peptides was validated in M/M patients. Proteotypic peptide concentrations correlated linearly with quantitation by non-Z and non-S peptides [slopes (Spearman correlation coefficient) of 1.09 (0.89) and 0.98 (0.80), respectively]. Allele-specific quantitation showed significant differences in wild-type protein expression in M/Z and M/S patients. Although average total A1AT concentration was lower for M/Z patients, the percentage of wild-type protein in M/Z patients was significantly higher at 82 % (55- > 95 %) compared to 63 % (43-83 %) for M/S heterozygotes. In a cohort of M/Z patients with sufficient total A1AT (≥80 mg/dL), half had insufficient wild-type protein that could have clinical implications for pulmonary dysfunction.

**Conclusions:**

For the first time, a method to quantitate A1AT allele protein expression is described. Given the wide range of circulating wild-type protein observed in heterozygous patients, this method has the potential to reveal correlations between allele concentration and development and/or severity of clinical symptoms.

## Background

Alpha-1-antitrypsin (A1AT) is a serine protease inhibitor that protects the lung from enzymatic damage [1]. Deficiency is caused by genetic mutations in the A1AT gene [2, 3]. Over 100 alleles have been identified spanning from fully expressed alleles that produce functional protein to null alleles that express no detectable A1AT [4]. The M allele encodes the wild-type protein and the Z and S alleles are the most common deficiency alleles. Both S and Z alleles result in reduced protein concentrations in circulation from decreased production (S allele) or polymerization and secretion blockage in hepatocytes (Z allele) [5–7]. The Z allele is expressed at lower circulating concentrations than the S allele [8]. Thus, Z/Z patients are at risk of pulmonary damage from proteolytic enzymes and can also present with liver sequela from hepatocyte damage [3].

Traditionally, laboratory analysis of A1AT deficiency involves two steps: (1) immuno-quantitation of serum A1AT protein and (2) identification of the disease-associated A1AT alleles [9–12]. Identification of A1AT alleles is routinely done by isoelectric focusing of serum proteins or molecular analysis of white blood cell DNA. The most recently described method for allele identification utilizes liquid chromatography and tandem mass spectrometry (LC-MS/MS) of serum [13]. This method identifies tryptic peptides from the S and Z portion of the protein to determine if the peptide has the Z or S mutation or if it is wild-type (non-Z, non-S). In the same assay, total A1AT quantitation can also be performed by detection of a proteotypic peptide that is common among all alleles, allowing for multiplexing of quantitation and allele identification in one analytical assay.

Patients who express both wild-type and mutant A1AT, namely M/Z and M/S heterozygotes, display a wide range of serum A1AT concentrations [8, 14]. However, few heterozygous patients express A1AT at concentrations that would raise concern for deficiency; the question of whether these individuals are at risk for clinical symptoms is controversial [15, 16]. Because current methods are only capable of quantitating total A1AT, the impact of the ratio of wild-type to mutant protein on clinical prognosis has not been probed. The ability to perform allele-specific quantitation may yield new biological and clinical insights about A1AT function and deficiency. Here, we demonstrate the capability of allele-specific quantitation in heterozygous patients by an LC-MS/MS method.

## Methods

### Reagents

Research-grade ammonium bicarbonate (NH_4_HCO_3_), trifluoroethanol (TFE), iodoacetamide (IAA), trifluoroacetic acid (TFA), and trypsin (T-1426) were purchased from Sigma-Aldrich; dithiothreitol (DTT) and formic acid from Fluka; fetal calf serum (FCS) from Invitrogen (Gibco 10437–028); and highly purified A1AT protein from Athens Research & Technology (16-16-011609). Water, acetonitrile, n-propanol, and dimethylformamide (DMF) were HPLC grade. Isotopically-labeled peptide standards were synthesized in-house; analytical HPLC showed purity to be >90 %. Stock peptide solutions were stored as 1 mg/mL in water and 0.1 % formic acid.

### Study population

A total of 368 serum samples (244 M/M, 61 M/Z, and 63 M/S) collected in serum separator tubes were obtained from individuals referred to the Mayo Clinic Immunology Laboratory for evaluation of possible A1AT deficiency. Samples were collected over several months after A1AT quantitation by immunoassay and phenotyping by electrophoresis. Samples were stored refrigerate up to 7 days or frozen up to 3 months prior to testing. All samples were submitted for A1AT quantification and phenotyping and were analyzed after approval and consent waiver by the Mayo Clinic Institutional Review Board (#09-001265).

### Trypsin digestion protocols

Lyophilized, purified human A1AT was dissolved in water at 260 mg/dL for use as standards. Patient serum samples (5 μL) or purified A1AT standards (appropriate dilutions of stock A1AT plus 5 μL of FCS) were added to 5 μL of 100 mM DTT and 25 μl labeled peptide standards (0.23 mg/mL in 100 mM NH_4_HCO_3_ buffer) and incubated for 30 min at 55 °C. 10 μL of 100 mM IAA was added and incubated for 60 min protected from light at room temperature. 25 μL of 1 mg/mL trypsin, 100 μL of 100 mM ammonium bicarbonate buffer, and 300 μL of deionized water were added and samples were placed in an indirect sonicator (Misonix, S-4000 MPX) and received one second pulses at half amplitude for 5 s. Samples were placed in an incubator for 30 min at 37 °C, followed by digestion termination with 10 μL of 10 % formic acid.

### LC-MS/MS analysis of patient serum samples

Sample analysis was performed using selective reaction monitoring (SRM) on an API 5000 triple quadrupole mass spectrometer (AB SCIEX) with an electrospray ionization source coupled to a TLX2 liquid chromatography system (Thermo, Cohesive). A Hypersil Gold C18, 50×3 mm, 5 μm particle size column (Thermo, 25005–053030) was used to separate the peptides in a water/acetonitrile (with 0.1 % formic acid) linear gradient at 0.25 mL/min.

One transition per peptide was monitored (adapted from reference [13]).

### Quantitation of total, wild-type and mutated A1AT protein

Quantitation of total and wild-type A1AT protein was performed by comparison of observed peak area ratios (peak height of unlabeled peptide divided by peak height of labeled peptide) to standard curves for the proteotypic, non-S, and non-Z peptides. The proteotypic peptide refers to a tryptic peptide which is constant across all alleles. This peptide is used for quantitation of total A1AT, and results should be consistent with nephelometry. The non-S peptide refers to the tryptic peptide containing the wild-type sequence at the location of the S mutation. In an M/S heterozygote, quantitation of this peptide reflects only the M allele in circulation. Analogously, the non-Z peptide is the tryptic peptide containing the wild-type sequence at the site of the Z mutation. In an M/Z heterozygote, quantitation of this peptide also reflects only the amount of the M allele present. The concentration of circulating S and Z protein in heterozygotes was then calculated indirectly from the measured total A1AT minus wild-type M protein.

Relative ratios of wild-type to total for each heterozygote were calculated. The ratio of wild-type protein to total A1AT in the two heterozygous cohorts was found to fit a Gaussian distribution in the probability plot using EP Evaluator so central 95^th^ percentile intervals of wild-type ratios were calculated using parametric analysis. (EP Evaluator release 10, Data Innovations, South Burlington, VT). Total A1AT concentration was found to be skewed within the different cohorts (SAS, v. 9.4 and the Wilcoxon signed rank test) so non-parametric Passing-Bablock regression was used (an R 3.1.1 function) to examine the association between LC-MS/MS results to total A1AT by immunoassay. Spearman correlation coefficient (ρ) was used to assess the strength of the correlation. Wilcoxon signed rank test for paired data was used to compare the amount of M protein to total A1AT in heterozygous patients.

### A1AT immunoassay

A1AT was quantified using immuno-nephelometry on a Behring Nephelometer II (Dade Behring) with commercially available reagents and standards (Siemens; A1AT reagent PSAZ15 and calibrator/standard OQIM15). The reference interval for A1AT in healthy subjects is 100–190 mg/dL and coefficient of variation for the assay is 5 %. All assays were performed according to manufacturer’s instructions.

## Results

### Measuring total A1AT concentrations

**Fig. 1:**
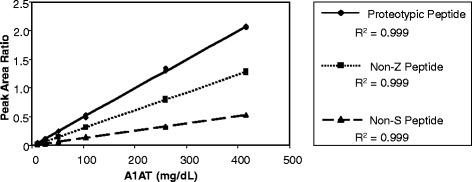
Standard curves generated using purified human A1AT protein. Standard curves for the proteotypic, non-Z and non-S peptides are shown. The standard curves cover concentrations ranging from 10 mg/dL to 416 mg/dL

**Fig. 2:**
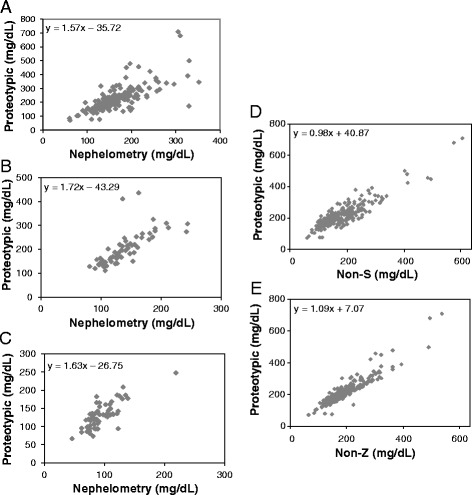
**a**. Comparison of quantitation of total A1AT in M/M homozygotes (*n* = 244). Total A1AT concentration by LC-MS/MS using the proteotypic peptide (y-axis) compared to the concentration measured by immuno-nephelometry (x-axis). **b**. Comparison of total A1AT by nephelometry and LC-MS/MS in M/S heterozygous patients (*n* = 63). **c**. Comparison of total A1AT by nephelometry and LC-MS/MS in M/Z heterozygous patients (*n* = 61). **d**. Total A1AT in the M/M cohort by LC-MS/MS using the proteotypic peptide (y-axis) compared to total A1AT by LC-MS/MS using the non-S peptide (x-axis). **e**. Total A1AT in the M/M cohort by LC-MS/MS using the proteotypic peptide (y-axis) compared to total A1AT by LC-MS/MS using the non-Z peptide (x-axis)

Standard curves for proteotypic, non-Z, and non-S peptides are shown in Fig. 1. Peak ratios for all three peptides demonstrated a linear response. Next, total A1AT was quantitated in a cohort of M/M homozygotes by LC-MS/MS using the proteotypic peptide and compared to results obtained by immunoassay (Fig. 2a). Similar to previous studies, the LC-MS/MS A1AT proteotype quantitation in homozygous M/M samples has a positive bias compared to the immunoassay (slope = 1.57 with 95 % confidence interval 1.49-1.66, Spearman ρ = 0.77) [13]. Similar comparisons of total A1AT concentration from LC-MS/MS compared to immunoassay in heterozygous M/S (slope = 1.72 with 95 % confidence interval 1.52-1.95, Spearman ρ = 0.88) and M/Z (slope = 1.63 with 95 % confidence interval 1.39-1.94, Spearman ρ = 0.74) sera yielded similar positive biases (Fig. 2b and c). In all cohorts the slope was greater than the y-intercept leading to an overall positive bias in the assay. A1AT concentrations in M/M serum samples were also measured using the non-S and non-Z peptides. Protein concentrations calculated using non-S and non-Z peptides compared to the proteotypic peptide are shown in Fig. 2d and e. Because there are no mutated alleles, the A1AT concentration calculated using each internal peptide should yield results in M/M homozygotes within the imprecision of the assay. Indeed, regression analysis shows that in homozygous M/M samples the LC-MS/MS method yields similar results when comparing the proteotypic and non-S or proteotypic and non-Z peptides (slope of 0.98 with 95 % confidence interval 0.90-1.08, Spearman ρ = 0.80 and slope of 1.09 with 95 % confidence interval 1.02-1.16, Spearman ρ = 0.89, respectively).

### Allele-specific quantitation

**Fig. 3:**
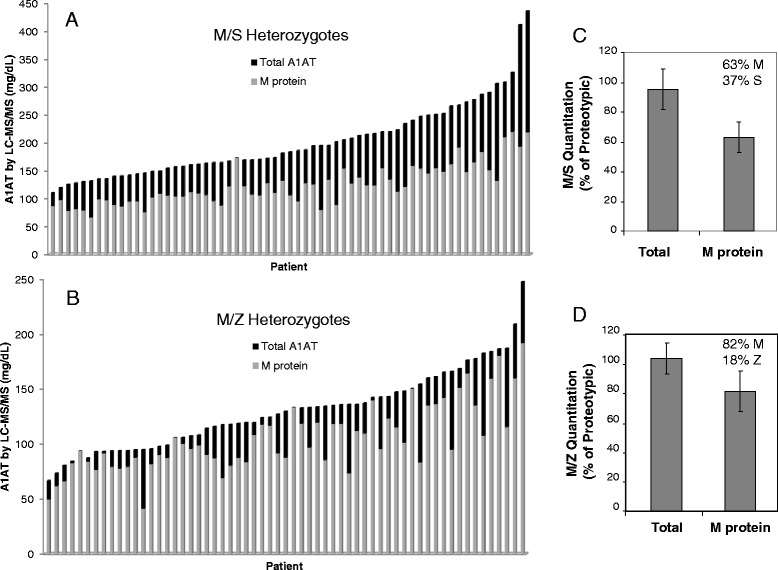
Allele-specific quantitation in M/S and M/Z heterozygotes. **a**. Total and wild-type A1AT were quantitated in M/S heterozygotes (*n* = 63) by LC-MS/MS. Total A1AT concentration determined from the proteotypic peptide (black bars) was compared to the wild-type M concentration as measured by the non-S peptide (gray bars). **b**. Total and wild-type A1AT were quantitated in M/Z heterozygotes (*n* = 61) by LC-MS/MS. Total A1AT concentration determined from the proteotypic peptide (black bars) was compared to the wild-type M concentration as measured by the non-Z peptide (gray bars). **c**. The concentration of non-S (M protein) and non-Z (total) in each M/S sample was expressed as percent of total A1AT concentration as measured by the proteotypic peptide (average ± 1 standard deviation are shown). **d**. The concentration of non-Z (M protein) and non-S (total) in each M/Z sample was expressed as percent of total A1AT concentration as measured by the proteotypic peptide (average ± 1 standard deviation are shown)

The LC-MS/MS method for A1AT allele-specific quantitation was performed in M/S and M/Z cohorts. As expected, in M/S and M/Z heterozygotes, the concentration of wild-type (non-S and non-Z, respectively) M protein was less than the concentration of total (proteotypic) A1AT protein (Fig. 3a and b) (*p* < 0.0001). Within the M/S heterozygote cohort, wild-type protein represented, on average, 63 % of total A1AT protein (Fig. 3c), with the remaining 37 % composed of mutant S protein (*p* < 0.0001). The central 95%ile range of wild-type protein in M/S heterozygotes was 43 to 83 %. Conversely, in M/Z heterozygotes, an average of 82 % of circulating A1AT protein was M protein and 18 % Z protein (*p* < 0.0001) with the central 95 % range of M protein being 55 to >95 % (Fig. 3d). There was a significantly higher percentage of M protein in M/Z heterozygous patients compared to M/S patients (*p* < 0.0001).

**Fig. 4:**
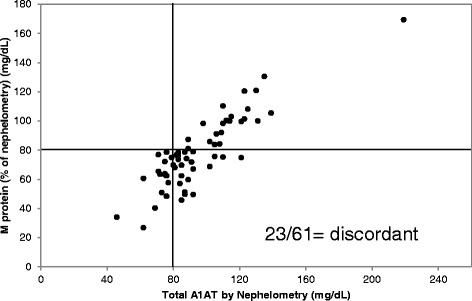
Clinical concordance of wild-type A1AT and total A1AT in 61 M/Z patients. Concentration of wild-type protein (as calculated from percent of nephelometry concentration), y axis, versus total A1AT concentration by nephelometry, x-axis, in M/Z heterozygotes

Lastly, whether M/Z patients express sufficient total A1AT by nephelometry but insufficient wild-type protein was assessed. The concentration of wild-type A1AT was calculated from the nephelometry result based on the percentage of wild-type protein established by mass spectrometry. This was then compared to the total A1AT concentration obtained by nephelometry. The Z protein has been reported to be six times less effective at inhibiting neutrophil elastase compared to wild-type protein [17]. Our data indicate that, on average, less than 20 % of A1AT in M/Z heterozygotes is Z protein so the activity of the Z protein is likely insignificant compared to the wild-type activity in these patients. The same assumptions cannot be made for the S protein so the analysis was performed for only the M/Z cohort. As shown in Fig. 4, out of 61 M/Z patients tested 38 (62.2 %) results were clinically concordant as defined by expressing sufficient (≥80 mg/dL) or insufficient (<80 mg/dL) A1AT by both nephelometry and LC-MS/MS [8]. However, of the 45 patients whose total A1AT was ≥ 80 mg/dL by nephelometry, 23 (51.1 %) expressed < 80 mg/dL wild-type protein by LC-MS/MS (Fig. 4, bottom right quadrant).

## Discussion

The relative risk for disease progression in non-smoking M/Z heterozygotes remains controversial [18–21]. A comparison between individuals in the Lung Health Study with either rapid or slow decline in forced expiratory volume per second (FEV_1_) indicated the M/Z phenotype was associated with rapid decline (odds ratio 2.8 [95 % CI: 1.2, 7.3], p = 0.03) [18]. Also, a longitudinal study showed a slightly greater annual decrease in FEV_1_ in M/Z individuals compared with M/M individuals (25 versus 21 mL/year, p = 0.048) [20]. The odds ratio for developing airflow obstruction was 1.3 ([95 % CI: 1.0, 1.7], p = 0.023) in M/Z heterozygotes versus M/M homozygotes, with the increased COPD risk possibly limited to those with a symptomatic Z/Z first-degree relative. In contrast, results from the Tucson Epidemiologic Study of Airways Obstructive Diseases failed to show any increased risk of developing airflow obstruction in M/Z heterozygotes [22]. Additionally, a meta-analysis highlighted the inconsistency of results from available publications [23], with studies using categorical outcomes generally showing an increased risk while those using continuous measures (eg, FEV_1_) failing to show an excess risk in M/Z heterozygotes. Most recently in a family-based study including relatives of M/Z probands diagnosed with COPD, M/Z subjects were found to have more airflow obstruction and a higher odds ratio for COPD compared to M/M subjects (odds ratio 5.18 [95 % CI:1.27,21.15]) [24]. The reduced function and higher risk in M/Z individuals was accentuated in those with a history of smoking compared to never-smokers.

The ability to measure allele-specific expression in heterozygous A1AT patients has the potential to probe questions of A1AT biology and disease progression. While we show the analysis can be performed in heterozygous carriers of the S allele, less than two percent of M/S samples sent for clinical testing have total A1AT <80 mg/dL and are thus unlikely to be at risk of developing symptoms of deficiency [8]. However, the spectrum of serum total A1AT protein concentrations in M/Z phenotypes is wide, ranging from 30–322 mg/dL with less than 30 % of those tested expressing total A1AT at < 80 mg/dL [8]. Given the small percentage expressing A1AT near the threshold for deficiency, it is not surprising that results from population studies for emphysema risk in non-smoking M/Z heterozygotes are contradictory. Until now, it has not been possible to determine if M/Z heterozygotes with low wild-type A1AT are at increased risk of clinical manifestations of deficiency, particularly pulmonary disease if challenged with environmental triggers such as smoking or hazardous work place air conditions, despite total A1AT ≥80 mg/dL. Current clinical A1AT protein analysis methods serve to quantify total A1AT protein and identify deficiency alleles. Gel phenotyping, although capable of allele identification, is not quantitative while immunoassays quantitate total A1AT regardless of allele identity. The data presented here on heterozygous patients demonstrate that LC-MS/MS is capable of selectively quantitating wild-type A1AT protein using methods already available at referral laboratories. The availability of this technique could become even more common at local hospitals as mass spectrometry becomes increasingly prevalent in routine practice.

Allele-specific quantitation by LC-MS/MS reveals that mutant S and Z alleles are found at lower circulating concentrations than wild-type protein in heterozygous patients (on average 37 % in an M/S cohort and 18 % in an M/Z cohort). These experimental findings correspond well with the known biological mechanisms of the two most common deficiency alleles. While both S and Z are deficiency alleles, patients with the Z/Z phenotype display less total circulating A1AT as compared to S/S patients (median of 25 ng/mL vs. 95 ng/mL, respectively) [8, 14]. This is also consistent with the disease pathogenesis in which the Z mutation causes decreased protein release from hepatocytes, while the S mutation is mostly released in circulation but at lower concentrations than the M allele [4–7]. This study is limited by the ability to only measure the M protein in heterozygous samples. Further assay development to allow for measurement of the S and Z proteins would likely improve quantitation accuracy. Another possible limitation could be measurement of circulating A1AT protein from patients with advanced liver injury sometimes observed in patients with A1AT deficiency. The suppression of all protein expression could complicate interpretation of allele-specific quantitation.

Augmentation therapy is the standard of care for pulmonary disease in patients with severe deficiency but generally discouraged for M/Z individuals given the lack of efficacy evidence [25]. To our knowledge, no studies have addressed whether the ratio of A1AT allele expression or the absolute concentration of wild-type protein correlates with pulmonary disease susceptibility in heterozygous patients or whether changes occur in allele expression over time that may correlate with disease progression. Results outlined in this study suggest that in heterozygous M/Z patients, significantly different ratios of wild-type to mutant protein exist. These differences cannot be predicted by total A1AT protein given that M/Z sera with less than 100 mg/dL of total A1AT as measured by nephelometry ranged from <50 % to > 95 % M protein. Additionally, our data show that half of M/Z heterozygous patients expressing sufficient total A1AT as measured by nephelometry express insufficient concentration of wild-type protein. It could be postulated that such differences may identify a subgroup of M/Z heterozygous patients that are more susceptible to spontaneous or environmentally induced pulmonary sequelae. Therefore, allele-specific A1AT quantitation by mass spectrometry opens the door to an entirely novel area for the study of A1AT biology and could be used to stratify patients into risk categories based on more information than allele identification and total A1AT concentration alone. Whether differential expression patterns within phenotype groups relate to susceptibility or severity of disease is under investigation.

## Conclusions

In conclusion, we show that independent quantitation of A1AT alleles in heterozygous patients is possible using LC-MS/MS. In addition, even though total A1AT protein is less in M/Z versus M/S patient, a greater percentage of circulating A1AT is wild-type in M/Z patients. Further, within the M/Z population there is wide variability in wild-type to mutant protein ratios in circulation. Some heterozygous M/Z individuals who have total A1AT >80 mg/dL, which would not be considered deficient, actually have <80 mg/dL of wild-type A1AT. Whether differential expression patterns within phenotype groups relate to susceptibility or severity of disease warrants further investigation.

## Abbreviations

A1AT: Alpha-1 antitrypsin
LD-MS/MS: Liquid chromatography tandem mass spectrometry
COPD: Chronic obstructive pulmonary disease
FEV: Forced expiratory volume
